# Characterization of Fruit and Seed Development in the Genera *Anacamptis* and *Serapias* (Orchidaceae)

**DOI:** 10.3390/plants14081229

**Published:** 2025-04-16

**Authors:** Emma Ortúñez, Alegría Pérez-Guillén, Roberto Gamarra

**Affiliations:** 1 Departamento de Biología, Universidad Autónoma de Madrid, 28049 Madrid, Spain; alegria.perez.guillen@gmail.com (A.P.-G.); roberto.gamarra@uam.es (R.G.); 2 Centro de Investigación en Biodiversidad y Cambio Global (CIBC-UAM), Universidad Autónoma de Madrid, 28049 Madrid, Spain; 3 Estación Experimental del Zaidín, Consejo Superior de Investigaciones Científicas, 18008 Granada, Spain

**Keywords:** ovary, placenta, ovule, embryo, testa, air space, raphides

## Abstract

Developmental changes in the anatomy along with the maturation from ovaries to fruits and ovules to seeds were analyzed in two terrestrial species of the related genera *Anacamptis* and *Serapias* (Orchideae, Orchidaceae), using light and scanning electron microscopy. Our results show that the proliferation of the placentae and the differentiation of the ovules are well developed at the beginning of the anthesis. After fertilization of the ovules, a cavity of free air space is formed surrounding the embryo, contributing to the later buoyancy of seeds in both genera. At the last days of their development, the seeds showed slanting ridges in the periclinal walls of the testa cells. Raphides were observed in the valves, formed by packed needles composed of calcium oxalate, which contribute to avoiding herbivory. Lignification observed in the endocarp cells of the placenta and in the testa cells can be related to protecting the ovules and embryos. Terrestrial orchids need a faster maturation to ensure the efficacy of fertilization due to seasonal environmental changes in temperate areas, so developmental changes during the maturation of fruits and seeds take place in a shorter time than in epiphytic orchids.

## 1. Introduction

There are many studies dealing with embryology in Orchidaceae, including micro- and megasporogenesis [1,2,3,4,5]. However, the developmental anatomy of ovaries and fruits has been much neglected in both terrestrial and epiphytic orchids. In the 1940s, Carlson [6] and Duncan and Curtis [7] published the first studies in the genera *Cypripedium* and *Paphiopedilum*, respectively, using light microscopy, but they lack continuity. In the 21st century, new studies focused on epiphytic orchids have revealed new results and methodologies [8,9,10], but there are many gaps in the study of terrestrial orchids, considering that the seed formation is a key factor to success in orchid propagation and conservation efforts [11].

A typical orchid ovary has three sterile and three fertile valves. Each fertile valve comprises two carpel halves and one marginal forked placenta projected toward the center of the unilocular ovary [6,12]. Along these valves, a large number of ovules are produced in the margin of the placentae. After pollination, pollen tubes continue to grow lengthwise within the cavity of the ovary, which has increased in size, and the ovules are ready for fertilization. Later, the pollen tubes degenerate, and the ovules and the ovary become seeds and mature fruit, respectively [9].

In Orchidaceae, the most common fruit is a fissuricidal capsule, with dehiscence lines separating the valves [12]. In the pericarp of the fruit, the lignification of the endocarp and the vascular bundles or the presence of mineral inclusions such as raphides and druses have been observed [8,13,14], contributing to the defense against herbivory.

The time from pollination to fertilization in orchids has been measured from 4 days to 10 months, usually longer in epiphytic orchids [15,16]. Studies about the anatomical development of ovaries and fruits have been carried out under controlled conditions and artificial pollination [6,8,9]. In these studies, the proliferation of the placentae and the differentiation of the ovules succeeded after the pollination, and developmental changes were related to the size variation in the fertile and sterile valves, the differentiation of the dehiscence regions, the input of pollen tubes, or the lignification of the pericarp [8,9].

After fertilization and the opening of the fruit, the mature seeds can be dispersed. Several studies have demonstrated the presence of large air spaces inside the seeds of terrestrial orchids, which contribute to their buoyancy during the seed dispersal by air [17,18]; however, the formation of the free air space has not been explained.

*Anacamptis* Rich. and *Serapias* L. are two of the most well-known terrestrial genera within the tribe Orchideae [19]. *Anacamptis* comprises 11 species distributed around Western Europe, North Africa, and Central Asia [20]. *Anacamptis morio* (L.) R.M. Bateman, Pridgeon and M.W. Chase, a representative species, is mainly found throughout temperate areas of Europe, extending from Southwestern Europe and Northern Africa to the Caucasus and surroundings of the Caspian Sea. *Serapias* comprises 17 species, reaching Western Europe, North Africa, and the Caucasus throughout the circum-Mediterranean countries. *Serapias lingua* L. is the most widespread species, extending from Portugal and northern Africa to Anatolia (Turkey) [21]. Both species often grow in populations with a large number of individuals. In the studied genera, mature seeds share the same micromorphological pattern, with slanting ridges in the periclinal walls [22,23,24], and a high percentage of free air space has been measured within them [22].

Due to the extremely scarce studies of ovary and fruit anatomy in terrestrial orchids, the present study documents the key anatomical events (development of placentae and ovules, maturation of seeds, development of free air space inside the seeds, formation of ornamentation pattern in the periclinal walls of the testa) during the formation of fruits and seeds in *Anacamptis* and *Serapias* to increase the knowledge of ovary and fruit developmental anatomy and contribute additional insight to plant development in general. In contrast to previous publications [6,8,9], this study was performed in the field, under non-controlled conditions and natural pollination of the specimens.

## 2. Results

### 2.1. Diameter and Length of the Ovaries

**Figure 1:**
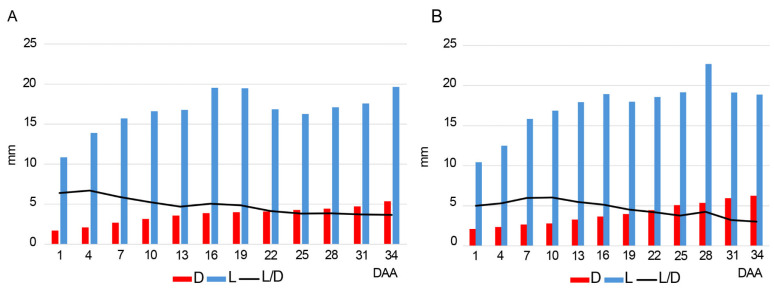
Average values of diameter (D, red color), length (L, blue color), and L/D ratio (line) of the ovaries (in mm) collected between 1 and 34 days after anthesis (DAA). (**A**) *Anacamptis morio*; (**B**) *Serapias lingua*.

In both species, the average value of the diameter increases over time from the anthesis to the ripening of the fruit. In the flowers collected at 1–7 DAA (days after anthesis), the diameter of the ovaries is always lower than 3 mm in both species. During the dehiscence of the fruits (31–34 DAA), the average value of the diameter is 5.34 mm in *Anacamptis morio* and 6.27 mm in *Serapias lingua* (Figure 1, Figures S1 and S2; Table S1). On the other hand, the length increases until 16 DAA, but it is more variable after this date (Figure 1, Figures S1 and S2; Table S1). In both species, the L/D ratio is higher in the first developmental days and usually decreases throughout maturation (Table S1).

### 2.2. Anatomical Development of the Ovary and the Pericarp

**Figure 2:**
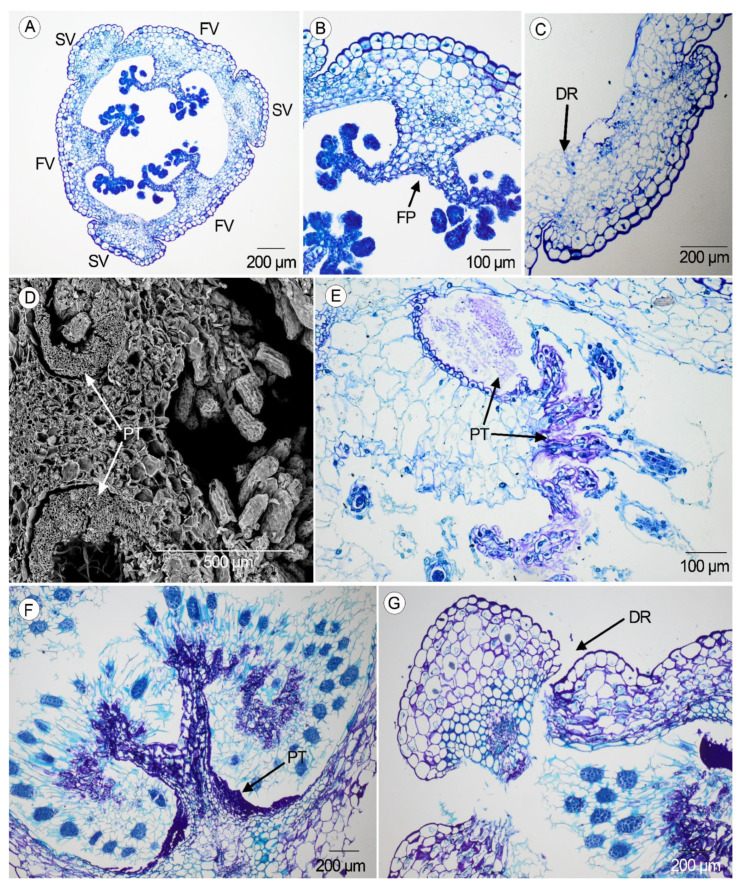
Developmental changes in the ovary in the studied species. (**A**) Cross-section of the ovary at 1–7 DAA in *A. morio* (Scale: 200 µm). (**B**) Detail of a sterile valve and a fertile valve with the forked placenta in *A. morio* (Scale: 100 µm). (**C**) Detail of the dehiscence region between sterile and fertile valves at 10–16 DAA in *S. lingua* (Scale: 200 µm). (**D**) Cross-section of the ovary at 19 DAA showing the large number of pollen tubes in *A. morio* under scanning electron microscope (Scale: 500 µm). (**E**) Cross-section at 19 DAA (black arrows indicate the pollen tubes and their extensions) in *A. morio* (Scale: 100 µm). (**F**) Detail of the forked placenta with the remnants of the pollen tube at 28 DAA in *S. lingua* (Scale: 200 µm). (**G**) Dehiscence of the fruit and detached sterile valve at 31–34 DAA in *S. lingua* (Scale: 200 µm). (SV—sterile valve, FV—fertile valve, FP—forked placenta, DR—dehiscence region, PT—pollen tube).

At the anthesis (1 DAA), the ovary of both species has three carpels divided into six valves, three fertile and three sterile, surrounding a free air space (Figure 2A). The outer epidermis of the ovary is a single layer with isodiametric cells provided with a thick wall. The internal tissue of the ovary has compact and isodiametric parenchyma cells, larger outward with thin walls, with a vascular bundle in the center of each valve. In the internal face of the fertile valves, the final branches of the placentae end up in ovules disposed to the free air space of the cavity. In the placenta region, a single layer of small and isodiametric cells with reinforced walls is observed. The three sterile valves are prominent (Figure 2B).

At 9–10 DAA, a dehiscence region starts to be distinguished between the sterile and the fertile valves as a layer of small cells with thin walls (Figure 2C).

In both species, the pollen tubes are visible inside the ovary at 19 DAA as a sign of successful pollination. In the transversal section, up to six pollen tubes run through a sinus at the sides of each forked placenta. Under a scanning electron microscope, each one of the pollen tubes is formed by a large number of tiny tubes (Figure 2D). These tubes extend and reach the ovules to fertilize them (Figure 2E, pollen tubes and extensions purple in color). In the following days, the pollen tubes begin to collapse (Figure 2F, collapsed pollen tubes dark purple in color). At 31–34 DAA, the seeds are mature, the sterile valves detach, and the fruit becomes dehiscent (Figure 2G).

**Figure 3:**
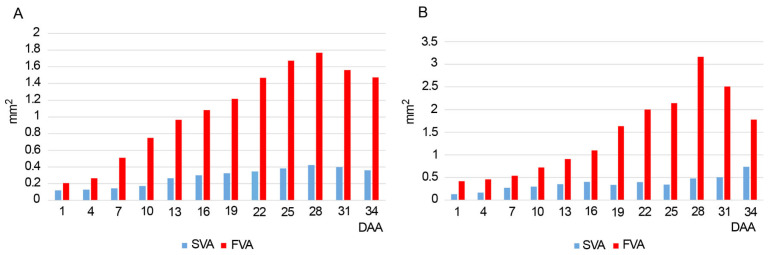
Average values of the sterile valve area (SVA) and fertile valve area (FVA) of the ovaries (in mm^2^) collected between 1 and 34 days after anthesis (DAA). (**A**) *Anacamptis morio*; (**B**) *Serapias lingua*.

From the 1st to the 28th DAA, both valves increase their area in the studied species, most markedly in the fertile valves. At 28–34 DAA, the areas tend to decrease due to the partial collapse of the pericarp cells (Figure 3, Figures S3 and S4; Table S2).

### 2.3. Ripening of Seeds

**Figure 4:**
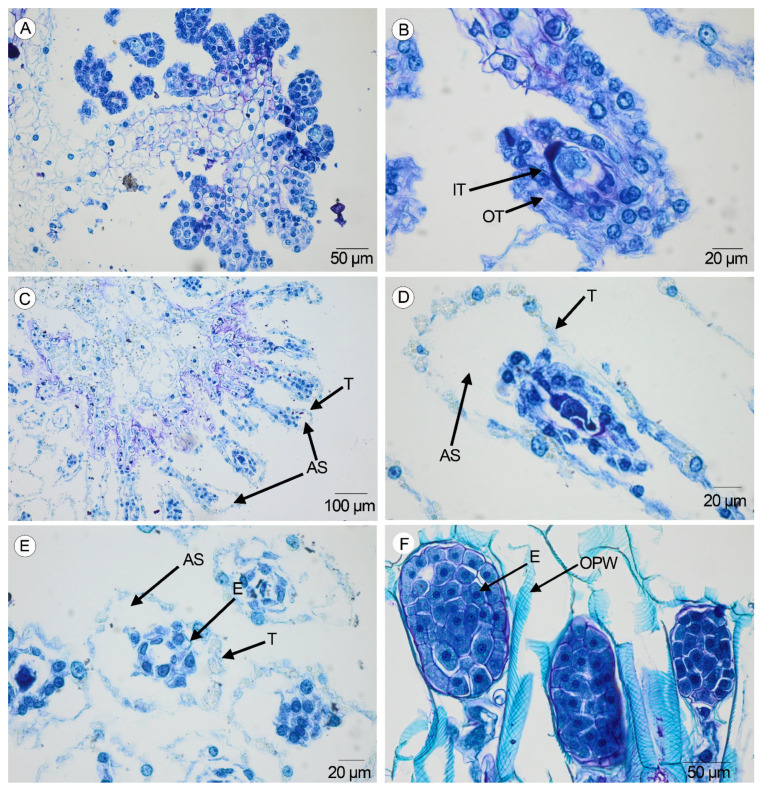
Developmental changes in the ovules and seeds in the studied species. (**A**) Globose ovules at 1–7 DAA in *A. morio* (Scale: 50 µm). (**B**) Bitegmic and anatropous ovule at 10–16 DAA in *A. morio* (Scale: 20 µm). (**C**) Cross-section of the fertile valve with immature seeds in *S. lingua* (Scale: 100 µm). (**D**) Longitudinal section of one immature seed at 19–28 DAA in *S. lingua* (Scale: 20 µm). (**E**) Cross-section of one immature seed at 19–28 DAA in *S. lingua* (Scale: 20 µm). (**F**) Longitudinal section of the seed with the multicellular embryo and the periclinal walls of the testa provided with ornamentation in *S. lingua* (Scale: 50 µm). (OT—outer tegument, IT—inner tegument, T—testa, AS—air space, E—embryo, OPW—ornamentation of the periclinal walls).

From the 1st to the 7th DAA, the ovules are globose with isodiametric cells in both species (Figure 4A). Later, the ovules become elongated, anatropous, and bitegmic (Figure 4B).

At 22 DAA, a free air space surrounding the embryo is clearly visible in the immature seeds of both species (Figure 4C). The inner integument degenerates completely, while the outer dehydrates and forms the testa, whose cells collapse partially, showing pronounced corners matched with the joints of the collateral cells, and part of the nucleus is observed in the periphery of the seed (Figure 4D–E). At 31–34 DAA, the ornamentation of the periclinal walls of the testa seeds is visible in both species (Figure 4F).

**Figure 5:**
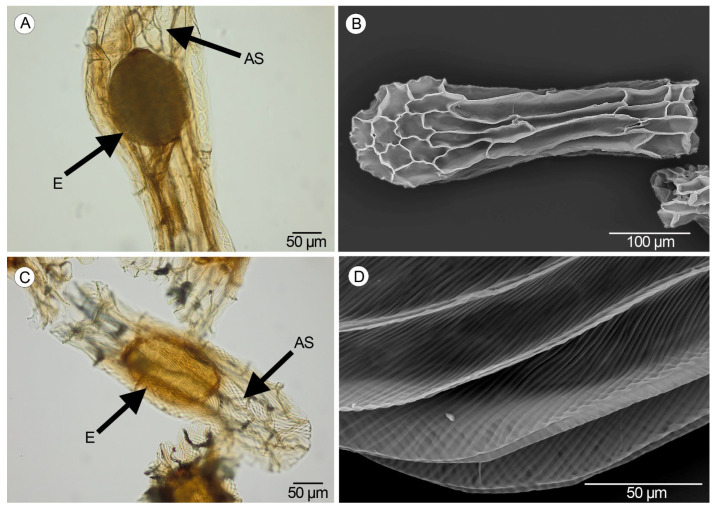
Mature seeds. (**A**) Seed of *Anacamptis morio* with the translucent testa and the internal free air space surrounding the embryo under light microscope (LM) (Scale: 50 µm). (**B**) Scanning electron micrograph of the seed with thin anticlinal walls and visible periclinal walls in *A. morio* (Scale: 100 µm). (**C**) Seed of *Serapias lingua* with the air space surrounding the embryo under LM (Scale: 50 µm). (**D**) Scanning electron micrograph showing the ornamentation pattern of the periclinal walls (slanting ridges) along the medial cells in the seeds of *S. lingua* (Scale: 50 µm). (AS—air space, E—embryo).

In both species, the mature seeds are fusiform in shape. The testa is formed by short and isodiametric polar cells with rectangular to elongated medial cells. Inside the testa, the embryo is surrounded by free air space (Figure 5A,C). Longitudinal and transversal anticlinal walls of the medial cells are straight and thin, and the periclinal walls are more visible. Cells of the apical pole show undulate anticlinal walls, which are straight in the cells of the basal pole (Figure 5B). In the periclinal walls, the ornamentation pattern formed by thin and slanting ridges is visible only when the seeds are mature (Figure 5D).

### 2.4. Lignification

**Figure 6:**
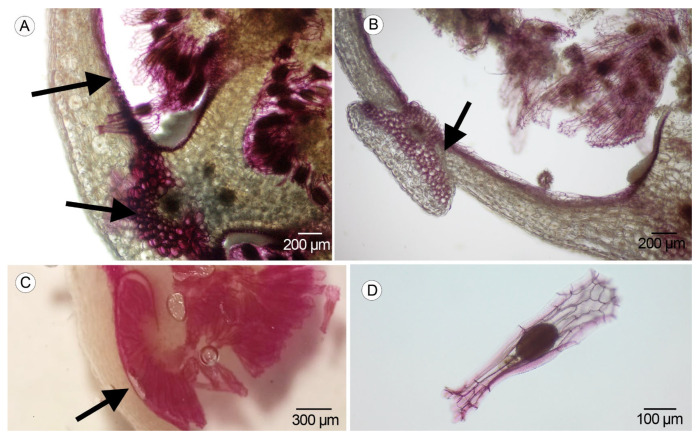
Phloroglucinol staining and lignification. (**A**) Cross-section of the mature ovary in *Serapias lingua* (black arrows indicate lignification in the vascular bundles and the external layer of the placenta) (Scale: 200 µm). (**B**) Cross-section of the mature ovary in *S. lingua* (black arrows show the dehiscence region) (Scale: 200 µm). (**C**) Cross-section of the ovary in *Anacamptis morio* (black arrow indicates the presence of lignin in the external later of the placenta) (Scale: 300 µm). (**D**) Mature seed of *S. lingua* with stained anticlinal and periclinal walls (Scale: 100 µm). In all the subfigures, the presence of lignin is stained purple with the phloroglucinol.

The process of lignification begins after the input of the pollen tubes. It was observed in the vascular bundles and in the endocarp cells of the placenta region from the 25th to the 34th DAA and in the anticlinal and periclinal walls of the testa cells in the mature seeds of both taxa at 31–34 DAA (Figure 6A–D).

### 2.5. Raphides

**Figure 7:**
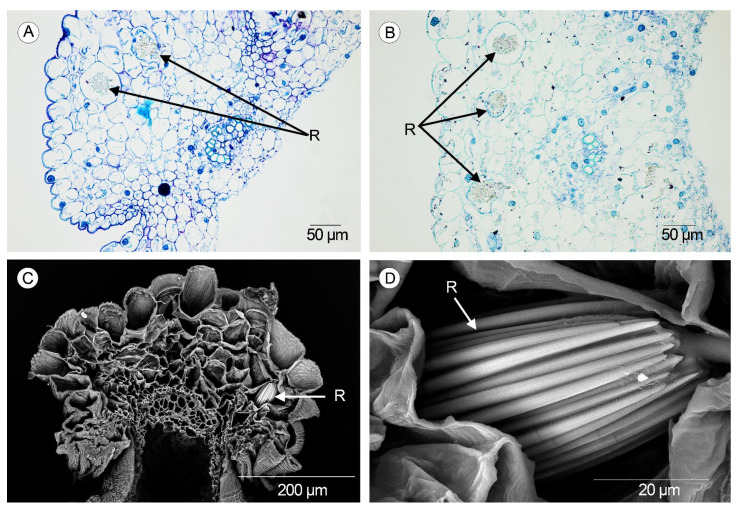
Raphides. (**A**) Idioblasts at the sterile valve in *Anacamptis morio* (Scale: 50 µm). (**B**) Idioblasts at the sterile and fertile valves in *Serapias lingua* (Scale: 50 µm). (**C**) Scanning electron micrograph of the cross-section of a sterile valve showing the raphides in *S. lingua* (Scale: 200 µm). (**D**) Scanning electron micrograph of the raphides formed by packed needles in *S. lingua* (Scale: 20 µm). (R—raphides).

Idioblasts containing raphides were observed in both types of valves in the species studied using LM and SEM (scanning electron microscope) (Figure 7A–C). Raphides consist of packed needles enclosed in the parenchyma of the mesocarp cells (Figure 7D). Depending on the orientation inside the idioblast, raphides can be observed as needles over the whole length or as polygons in cross-section. The elemental composition of the raphides was analyzed, and the EDS (energy-dispersive X-ray spectroscopy) shows calcium, carbon, and oxygen as the main chemical elements.

A summary of the developmental changes observed and the characterization of the structures analyzed is provided in Table 1.

## 3. Discussion

Our study is the first using individuals from wild populations of terrestrial taxa of the family Orchidaceae under non-controlled conditions of growing and pollination, unlike previous studies [6,7,8,9].

We observed a progressive increase in the diameter from ovaries to fruits, in concordance with previous data based on epiphytic orchids [8,25]. The increase in the diameter results mainly from the increased area of the fertile valves [9].

At 1 DAA, the division of the placentae in dichotomous branches and the formation of globose ovules was observed in both species, and the ovary shows a hollow area inside, in contrast to the epiphytic orchids, in which these processes happened after pollination [8,26]. The early presence of a hollow area enables the growth of the pollen tubes, which run throughout the unilocular cavity of the ovary toward the ovules, where the fecundation is carried out.

In our study, the dehiscence region between the sterile and fertile valves became apparent at 9–10 DAA, corresponding in time with the elongation of the ovules, which are anatropous and bitegmic, processes which succeed after pollination in epiphytic orchids [8,9,26]. The pollen tubes were visible inside the ovary at 19 DAA. Natural pollination was not registered but probably succeeded in the days immediately prior to the growth of the pollen tubes inside the ovary [26,27]. During the next days, the ripening process of the fruits and the maturation of seeds occurred, matching with the dehiscence between the valves, a similar period recorded by Guignard [15]. The results of Mayer et al. [8] and Dirks-Mulder et al. [9] show a longer developmental process of maturation of the fruits in epiphytic orchids than that in the terrestrial orchids in our study (until 110 days after pollination vs. 34 days after anthesis, respectively). Probably, the terrestrial orchids need a faster maturation to ensure the efficacy of the fertilization due to the seasonal environmental changes in temperate areas, so several key anatomical events, such as the dichotomic division of the placentae, the formation of ovules, the dehiscence region between the sterile and fertile valves, and the maturation of seeds takes place in a short time.

Regarding the valves, our study agrees with Dirks-Mulder et al. [9], because the increasing of the diameter of the ovaries is mainly caused by the increase in the fertile valves’ area. Our results show that the fertile valve areas are greater than the sterile valve areas until the ripening of the fruits, when a decrease is observed in both species due to the partial collapse of the pericarp cells, in concordance with the studies on epiphytic orchids [8,9].

Another change in the ripening from ovules to seeds is related to the formation of the free air space inside the seed between the testa and the embryo, as was mentioned by Carlson [6] in the genus *Cypripedium*. Air space is a common trait in seeds of terrestrial orchids [17,28], in contrast to the epiphytic ones, in which the embryos are encapsulated within the testa and the air space is smaller [8,29]. A large air space contributes to the buoyancy of seeds, related to the possibility of achieving long distances at dispersal [18,30]. Matching with the ripening of seeds in the last three days, an ornamentation pattern developed in the periclinal walls of the testa cells. In *Anacamptis morio* and *Serapias lingua*, it is formed by thin, slanting ridges. The sculpturing of the testa cells contributes to trapping air bubbles, improving the buoyancy in terrestrial orchids [30]. The formation of a large free air space and the sculpturing of the highly visible periclinal walls in the seeds are two relevant traits related to the terrestrial habit in many orchids [22,24,28] in contrast to the lower free air space and the narrowly to not-visible periclinal walls in the seeds of epiphytic orchids [17,24,31]. The ornamentation pattern is a good taxonomic trait in the study of seeds in the tribe Orchideae [32]. Our results, based on the anatomical development of the ovaries and the maturation of seeds, support that *Anacamptis* and *Serapias* are closely related genera [33,34].

The formation of the free air space surrounding the embryo and the development of ornamentation in the testa cells of terrestrial orchids are probably related to the buoyancy during seed dispersal.

Lignin stained with phloroglucinol was observed in both species at the 25th DAA in the endocarp cell on each side of the fertile valves, and at 31–34 DAA in the walls of the seed coat and the vascular bundles, in concordance with Mayer et al. [8], who observed lignified cells in the single layer of the endocarp and in the outer integument of the ovule. Using the same methodology, Dirks-Mulder et al. [9] also observed lignification in the endocarp cells around 10 weeks after pollination in the epiphytic orchid *Erycina pusilla* (L.) N.H.Williams & M.W. Chase and in the terrestrial *Epipactis helleborine* (L.) Crantz, but not in *Cynorkis fastigiata* Thouars, revealing different lignification patterns. Our observations in *Anacamptis morio* and *Serapias lingua* show similar patterns in both, with an evident lignification in the endocarp cells. That underscores the importance of increasing the studies in most epiphytic and terrestrial species, with the aim to know the variation in the ripening of fruits. The lignification process can be related to the protection of ovules and seeds against external agents.

Süngü et al. [14] observed raphides in the fruits of *Anacamptis* and cited prismatic crystals in *Serapias orientalis* (Greuter) H.Baumann & Künkele. We observed raphide crystals in the form of compact, packed needles within the cells of the mesocarp along the fertile and sterile valves. Our analysis using energy-dispersive X-ray spectroscopy showed a chemical composition dominated by calcium, carbon, and oxygen, in concordance with the composition of calcium oxalate [35], which contributes to avoiding herbivory [36]. Within Orchidaceae, raphides have also been cited in fruits and ovaries of the genus *Vanilla* Plum. ex Mill. [37,38] and in distinct genera within Epidendroideae [8,13], but they are more common in vegetative structures [39,40,41,42].

## 4. Materials and Methods

### 4.1. Plant Material and Fixation

**Figure 8:**
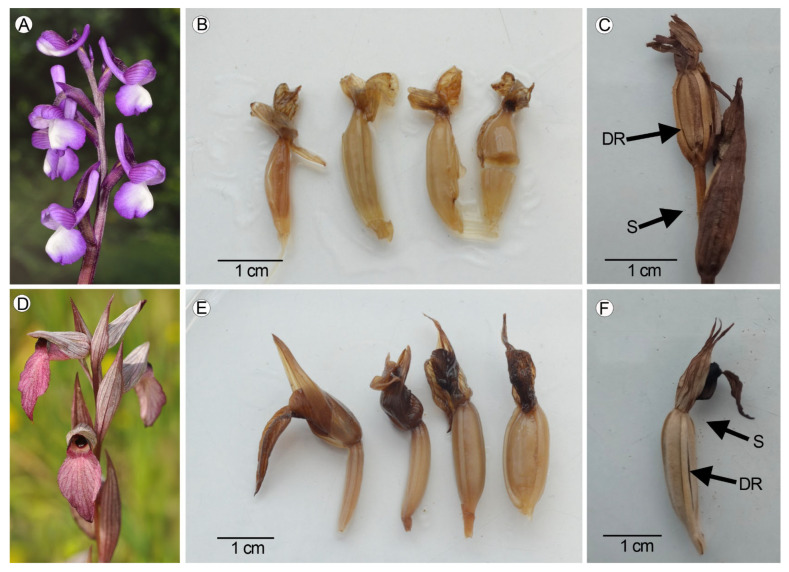
*Anacamptis morio*: (**A**) Inflorescence. (**B**) Ovaries collected at 4, 10, 19, and 25 DAA. (**C**) Dehiscent fruit and seeds collected at 34 DAA. *Serapias lingua*: (**D**) Inflorescence. (**E**) Ovaries collected at 4, 10, 19, and 25 DAA. (**F**) Dehiscent fruit and seeds collected at 34 DAA. (DR—dehiscence region, S—seeds).

Ovaries and fruits were collected in the field during May and June 2021 from two populations of *Anacamptis morio* (Figure 8A) and *Serapias lingua* (Figure 8D), separated by a distance of about 2 km, growing under non-controlled conditions in the locality of El Boalo, Community of Madrid (40°42′37″ N and 3°55′25″ W), central Spain. Both species grow in acid soils (granite) in seasonally wet areas in sunny meadows among patches of open forests dominated by *Quercus rotundifolia* Lam. (Fagaceae), with isolated individuals of *Fraxinus angustifolia* Vahl (Oleaceae). The average temperature during the months of May and June 2021 was 14.8 °C and 20.1 °C, and the average rainfall was 57 mm and 28 mm, respectively. During the field work, the daylight hours varied between 12 and 13 (data obtained from www.aemet.es, accessed on 11 september 2021). Populations were previously isolated with a perimeter fence to avoid herbivory from sheep and goats. Voucher specimens of the two studied species were deposited in the herbarium of the Universidad Autónoma de Madrid, Spain (MAUAM).

From the 1st day after anthesis (DAA), a permanent peg was situated near each specimen with the date of the beginning of the anthesis. Every three days, the ovary from the most basal flower of the inflorescence of three specimens was collected. A total of 36 ovaries/fruits of each species were collected at 1, 4, 7, 10, 13, 16, 19, 22, 25, 28, 31, and 34 DAA (Figure 8B,C,E,F).

Samples of ovaries and fruits were fixed in the field with formalin aceto-alcohol solution (FAA: 50% ethanol; 5% glacial acetic acid; 5% formalin; 40% distilled water) for 24 h at room temperature and finally stored in 70% ethanol at 5 °C. Photographs of the ovaries were taken with a Reflex Nikon D3200 (Nikon Corporation, Tokyo, Japan).

For each sample, ovary length and diameter were measured with a caliper (Mitutoyo Absolute Digimatic CD-15DCX, Mitutoyo Corporation, Kawasaki, Japan). Average values of length and diameter were calculated for each group of three samples collected in the days mentioned above. Graphics were created using R package ggplot2 v.3.5.

Ovaries were dissected transversely in sections for further studies under a light microscope (LM) and scanning electron microscope (SEM).

### 4.2. Microscopic Study

For LM studies, cross-sections were embedded in paraffin wax according to [12], and serial sections of 5 µm thickness were cut on a rotary microtome (Microm HM355 S, Thermo Fisher Scientific, Walldorf, Germany) and placed on a microscope slide. The slides were dewaxed and hydrated in a graded ethanol series (100%, 96%, 70%, 50%), stained with a solution of 0.2% Toluidine Blue and 0.2% Borax in distilled water, rehydrated with an ethanol series (50%, 70%, 96%, 100%), rinsed with distilled water, and mounted in Entellan^®^ synthetic resin (Merck, Darmstadt, Germany). The sections were examined, and images were taken using a light microscope Olympus BX41 (Olympus Corporation, Tokyo, Japan) with digital camera ColorView1 (Spectrographic Limited, Leeds, UK) using the software CellSens ver. 1.4. Later, the area of the fertile and sterile valves was measured following Dirks-Mulder et al. [9] to evaluate its variation along the development. Average values of sterile valve area (SVA) and fertile valve area (FVA) were calculated for each group of three samples collected in the days mentioned above. Graphics were created using R package ggplot2 ver. 3.5.

For each species, cross-sections of ovaries were cut using a scalpel. Later, they were stained with phloroglucinol-HCL solution to test for the presence of lignin in the cell walls under LM following the methodology in Dirks-Mulder et al. [9].

To analyze the testa and embryo of mature seeds under LM, a set of seeds was mounted with polyvinyl alcohol (PVA) following the methodology in Galán et al. [28].

For SEM analysis, transversal sections were dehydrated in a graded ethanol series and embedded in Hexamethyldisilazane (Aldrich, Merck, Darmstadt, Germany) for 24 h. In addition, seeds of the two species were obtained from mature capsules. Cross-sections and seeds were mounted on SEM stubs, coated with gold in a sputter-coater (SEM Coating System, Bio-Rad SC 502, Bio-Rad Laboratories, Madrid, Spain), and observed using a Philips XL30 (Philips, Amsterdam, The Netherlands) with an accelerating voltage of 20 kV. Qualitative data such as seed shape, morphology of the testa cells, periclinal and anticlinal walls, and the presence of ornamentation were studied following Gamarra et al. [23]. The chemical composition of specific observed structures was determined with energy-dispersive X-ray spectroscopy (EDS) using an Oxford INCAx-sight EDS detector (Oxford Instruments, High Wycombe, UK). This work was carried out in the “Servicio Interdepartamental de Investigación” (SIdI) at the Universidad Autónoma de Madrid, Spain.

We followed the terminology used in anatomical descriptions of Orchidaceae [8,9,12].

## 5. Conclusions

This study about the development from ovary to fruit and from ovules to seeds in terrestrial orchids improved the knowledge of the anatomical events in these structures, which contributes to conservation programs for terrestrial orchids. One of the highlights is the discovery that the proliferation of the placentae and the presence of a gap inside the ovary were formed at the first day after the anthesis, in contrast with epiphytic orchids. Furthermore, the gap between the embryo and the testa begins to develop after the input of the pollen tubes. This gap of air space is essential for the future buoyancy of the seeds, in contrast with epiphytic orchids, in which the free air space is less developed. Further studies including terrestrial orchids from different environmental conditions are needed to better understand the plant development.

## Figures and Tables

**Table 1:** Main developmental changes observed in the anatomy from ovary to fruit and ovules to seeds in *Anacamptis morio* and *Serapias lingua*.

Range of DAA	Ovary to Fruit	Ovules to Seeds
1–7	Increase in length and diameter Increase in the valves area Proliferation of placentae	Globose primordial ovules
10–16	Increase in length and diameter Increase in the valves area Beginning of the differentiation of the dehiscence region between sterile and fertile valves	Elongation of the ovules, becoming anatropous and bitegmic
19–28	Increase in diameter Increase in the valves area (principally, fertile valves) Input of the pollen tubes Beginning of the lignification of the endocarp cells in the placenta region Lignification of vascular bundles	Differentiation of the testa in the immature seeds Formation of the air space between embryo and testa
31–34	Increase in diameter Pollen tubes collapse Full lignification of the endocarp cells in the placenta region and vascular bundles Decrease in the valves area Sterile valves detached Dehiscence of the fruit	Full development of the embryo and the air space surrounding it Lignification of the walls in the testa seed Ornamentation of the periclinal walls

## Data Availability

All data generated or analyzed in this study are included in this published article.

## Supplementary Materials

The following supporting information can be downloaded at: https://www.mdpi.com/article/10.3390/plants14081229/s1, Figure S1: (**A**) Box plot of measurement of diameter; (**B**) length between 1 and 34 days after anthesis (DAA) in *Anacamptis morio*. Figure S2: (**A**) Box plot of measurement of diameter; (**B**) length between 1 and 34 days after anthesis (DAA) in *Serapias lingua*. Figure S3: (**A**) Box plot of measurement of sterile valve area (SVA); (**B**) fertile valve area (FVA) between 1 and 34 days after anthesis (DAA) in *Anacamptis morio*. Figure S4: (**A**) Box plot of measurement of sterile valve area (SVA); (**B**) fertile valve area (FVA) between 1 and 34 days after anthesis (DAA) in *Serapias lingua*. Table S1: Length (L), diameter (D), and L/D ratio of the ovaries/fruits collected between 1 and 34 days after anthesis (DAA) in *Anacamptis morio* and *Serapias lingua*. Table S2. Sterile valve area (SVA) and fertile valve area (FVA) of the ovaries and fruits collected between 1 and 34 days after anthesis (DAA) in *Anacamptis morio* and *Serapias lingua*.
