# Supplementary material for: Characterization of Fruit and Seed Development in the Genera Anacamptis and Serapias (Orchidaceae)
Source: Plants (Basel). 2025 Apr 16;14(8):1229. doi: 10.3390/plants14081229 (PMC12030694; doi:10.3390/plants14081229)
Supplement: Supplementary file 1 [file plants-14-01229-s001.zip › Table S1-ortunez-marzo25 1.pdf]

Table S1: Length (L), diameter (D) and L/D ratio of the ovaries/fruits collected between 359 1-34 days after anthesis (DAA), in Anacamptis morio and Serapias lingua

Species	DAA	L (mm)	D (mm)	L/D (mm)
A. morio	1	10.81	1.69	6.3964
		10.74	1.66	6.4698
		11.03	1.75	6.3028
	**Mean-1**	**10.86**	**1.7**	**6.39**
	4	13.75	1.98	6.9444
		13.56	2.07	6.5507
		14.38	2.19	6.5662
	**Mean-4**	**13.89**	**2.08**	**6.69**
	7	15.78	2.56	6.1640
		15.92	2.65	6.0075
		15.44	2.81	5.4946
	**Mean-7**	**15.71**	**2.67**	**5.89**
	10	16.97	3.15	5.3873
		16.51	3.24	5.095
		16.30	3.09	5.275
	**Mean-10**	**16.59**	**3.16**	**5.25**
	13	16.54	3.51	4.712
		16.92	3.63	4.661
		16.82	3.58	4.698
	**Mean-13**	**16.76**	**3.57**	**4.69**
	16	19.44	3.79	5.129
		18.43	3.85	4.787
		20.73	3.97	5.221
	**Mean-16**	**19.53**	**3.87**	**5.04**
	19	20.1	3.98	5.050
		19.56	4.01	4.877
		18.73	4.02	4.659
	**Mean-19**	**19.46**	**4.01**	**4.86**
	22	17.96	4.06	4.423
		17.65	4.08	4.325
		14.97	4.11	3,642
	**Mean-22**	**16.86**	**4.08**	**4.13**
	25	15.78	4.23	3.730
		16.73	4.27	3.918
		16.25	4.29	3.787
	**Mean-25**	**16.25**	**4.26**	**3.81**
	28	16.94	4.31	3.930
		17.21	4.41	3.902
		17.16	4.63	3.706
	**Mean-28**	**17.10**	**4.45**	**3.84**
	31	17.40	4.79	3.632
		17.28	4.66	3.708
		18.03	4.71	3.828
	**Mean-31**	**17.57**	**4.72**	**3.72**
	34	16.57	5.07	3.268
		20.79	5.28	3.937
		21.60	5.68	3.802
	**Mean-34**	**19.65**	**5.34**	**3.67**

S. lingua	1	10.58	2.10	5.038
		10.47	2.03	5.157
		10.28	2.14	4.803
	**Mean-1**	**10.44**	**2.09**	**5.00**
	4	12.17	2.35	5.178
		12.48	2.29	5.449
		12.86	2.41	5.336
	**Mean-4**	**12.50**	**2.35**	**5.32**
	7	13.36	2.59	5.158
		15.31	2.62	5.843
		18.83	2,73	6,897
	**Mean-7**	**15.83**	**2.65**	**5.97**
	10	19.56	2.77	7.061
		14.60	2.81	5.195
		16.50	2.83	5.830
	**Mean-10**	**19.89**	**2.80**	**6.03**
	13	18.09	3.37	5.367
		17.56	3.25	5.403
		18.13	3.19	5.683
	**Mean-13**	**17.93**	**3.27**	**5.48**
	16	17.72	3.51	5.048
		20.26	3.77	5.374
		18.85	3.74	5.040
	**Mean-16**	**18.94**	**3.67**	**5.15**
	19	16.00	3.91	4.092
		21.58	3.99	5.408
		16.35	4.00	4.087
	**Mean-19**	**17.98**	**3.97**	**4.53**
	22	17.94	4.37	4.105
		19.1	4.41	4.331
		18.65	4.52	4.126
	**Mean-22**	**18.56**	**4.43**	**4.19**
	25	16.10	5.01	3.213
		17.59	5.10	3.449
		23.81	5.13	4.641
	**Mean-25**	**19.17**	**5.08**	**3.77**
	28	22.07	5.16	4.277
		24.15	5.48	4.406
		21.89	5.42	4.038
	**Mean-28**	**22.70**	**5.35**	**4.24**
	31	18.89	5.82	3.245
		18.96	6.06	3.128
		19.56	5.95	3.287
	**Mean-31**	**19.13**	**5.94**	**3.22**
	34	18.82	6.19	3.040
		18.13	6.3	2.877
		19.73	6.31	3.126
	**Mean-34**	**18.89**	**6.27**	**3.01**
