# Supplementary material for: Characterization of Fruit and Seed Development in the Genera Anacamptis and Serapias (Orchidaceae)
Source: Plants (Basel). 2025 Apr 16;14(8):1229. doi: 10.3390/plants14081229 (PMC12030694; doi:10.3390/plants14081229)
Supplement: Supplementary file 1 [file plants-14-01229-s001.zip › Table S2-ortunez-marzo25 1.pdf]

Table S2. Sterile valve area 360 (SVA) and fertile valve area (FVA) of the ovaries and fruits collected between 1-34 days after anthe-361 sis (DAA) in Anacamptis morio and Serapias lingua

Species	DAA	SVA (mm^2)	FVA (mm^2)
A. morio	1	0.12009	0.20768
		0.12037	0.20741
		0.11814	0.20646
	**Mean-1**	**0.11953**	**0.20718**
	4	0.12762	0.26531
		0.12697	0.26513
		0.12797	0.26389
	**Mean-4**	**0.12752**	**0.26477**
	7	0.13796	0.51062
		0.14331	0.50998
		0.14285	0.51173
	**Mean-7**	**0.14316**	**0.51077**
	10	0.17316	0.74739
		0.17304	0.75037
		0.17236	0.74674
	**Mean-10**	**0.17285**	**0.74816**
	13	0.26427	0.96430
		0.26379	0.96512
		0.26440	0.96219
	**Mean-13**	**0.26415**	**0.96387**
	16	0.30108	1.08205
		0.30181	1.09107
		0.30088	1.07378
	**Mean-16**	**0.30125**	**1.08230**
	19	0.32495	1.21571
		0.32718	1.22193
		0.32539	1.21349
	**Mean-19**	**0.32584**	**1.21704**
	22	0.34612	1.46742
		0.34911	1.46763
		0.34462	1.46612
	**Mean-22**	**0.34661**	**1.46705**
	25	0.38153	1.67210
		0.38417	1.68723
		0.37851	1.66128
	**Mean-25**	**0.38140**	**1.67353**
	28	0.42444	1.76763
		0.42395	1.77428
		0.42134	1.76327
	**Mean-28**	**0.42324**	**1.76839**
	31	0.39720	1.56122
		0.40075	1.57087
		0.39584	1.55405
	**Mean-31**	**0.39793**	**1.56206**
	34	0.36199	1.47184
		0.36147	1.47920
		0.36015	1.46831
	**Mean-34**	**0.36120**	**1.47311**

S. lingua	1	0.12962	0.41517
		0.13429	0.41597
		0.12717	0.41484
	**Mean-1**	**0.13036**	**0.41532**
	4	0.16763	0.45909
		0.16779	0.45923
		0.16534	0.45769
	**Mean-4**	**0.16705**	**0.45867**
	7	0.27182	0.53908
		0.27385	0.54003
		0.27104	0.53685
	**Mean-7**	**0.27224**	**0.53865**
	10	0.29686	0.71908
		0.29684	0.71968
		0.29583	0.71645
	**Mean-10**	**0.29651**	**0.71840**
	13	0.35287	0.90689
		0.35269	0.89740
		0.35305	0.91329
	**Mean-13**	**0.35287**	**0.90586**
	16	0.40426	1.09697
		0.40407	1.08836
		0.40334	1.10229
	**Mean-16**	**0.40389**	**1.09587**
	19	0.33884	1.63180
		0.33911	1.64059
		0.33746	1.62401
	**Mean-19**	**0.33847**	**1.63213**
	22	0.39624	1.99963
		0.40013	2.00177
		0.39321	1.99386
	**Mean-22**	**0.39652**	**1.99842**
	25	0.34124	2.14848
		0.34188	2.10633
		0.34117	2.16894
	**Mean-25**	**0.34143**	**2.14125**
	28	0.47951	3.15328
		0.48067	3.19501
		0.47836	3.14571
	**Mean-28**	**0.47951**	**3.16466**
	31	0.50326	2.51709
		0.50076	2.47307
		0.50407	2.53769
	**Mean-31**	**0.50269**	**2.50928**
	34	0.73644	1.76982
		0.73958	1.81046
		0.73637	1.75408
	**Mean-34**	**0.73746**	**1.77812**
