# Supplementary figures and images for: Characterization of Fruit and Seed Development in the Genera Anacamptis and Serapias (Orchidaceae)
Source: Plants (Basel). 2025 Apr 16;14(8):1229. doi: 10.3390/plants14081229 (PMC12030694; doi:10.3390/plants14081229)

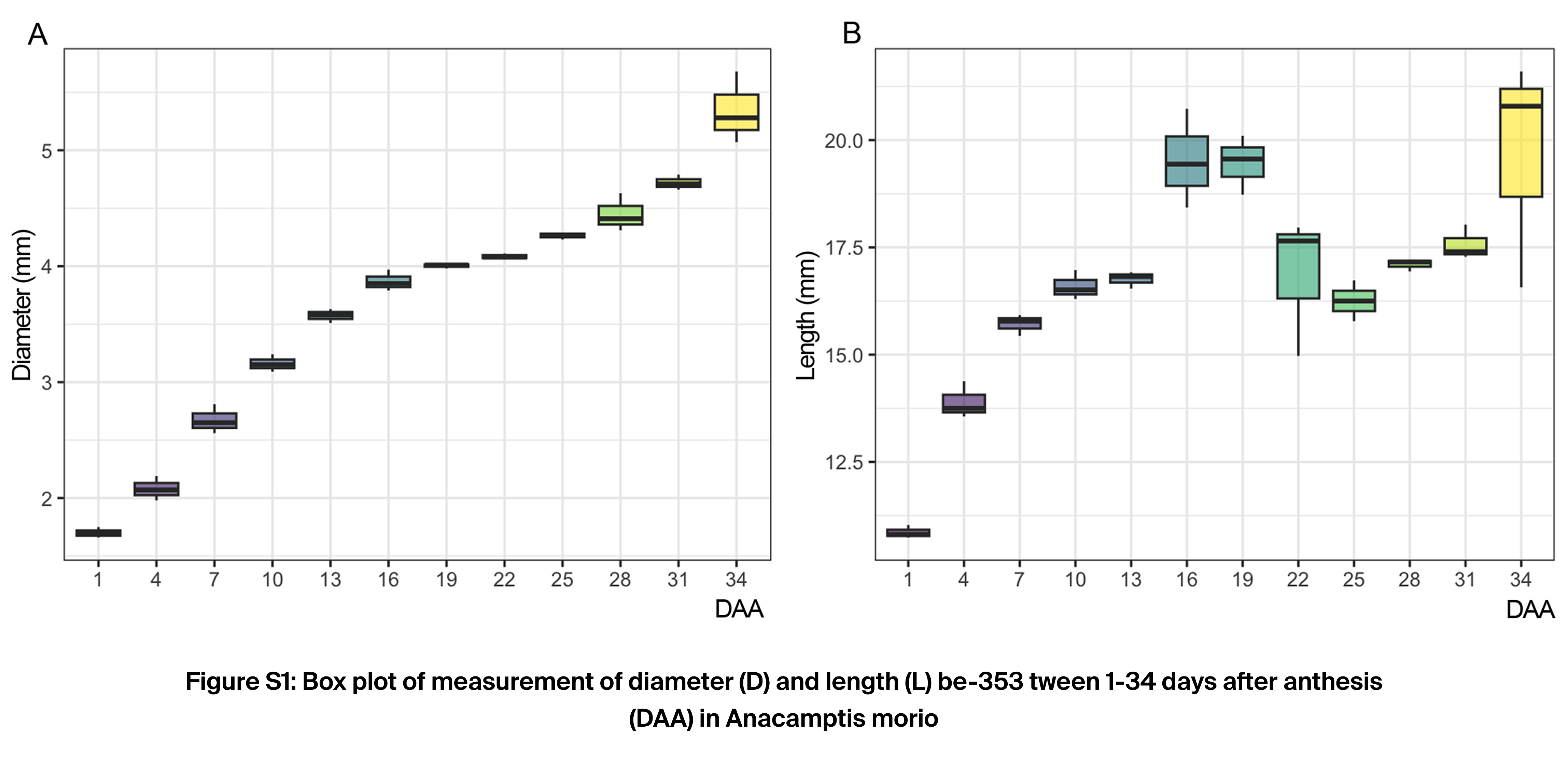

Supplement: Supplementary file 1 [file plants-14-01229-s001.zip › S1.png]

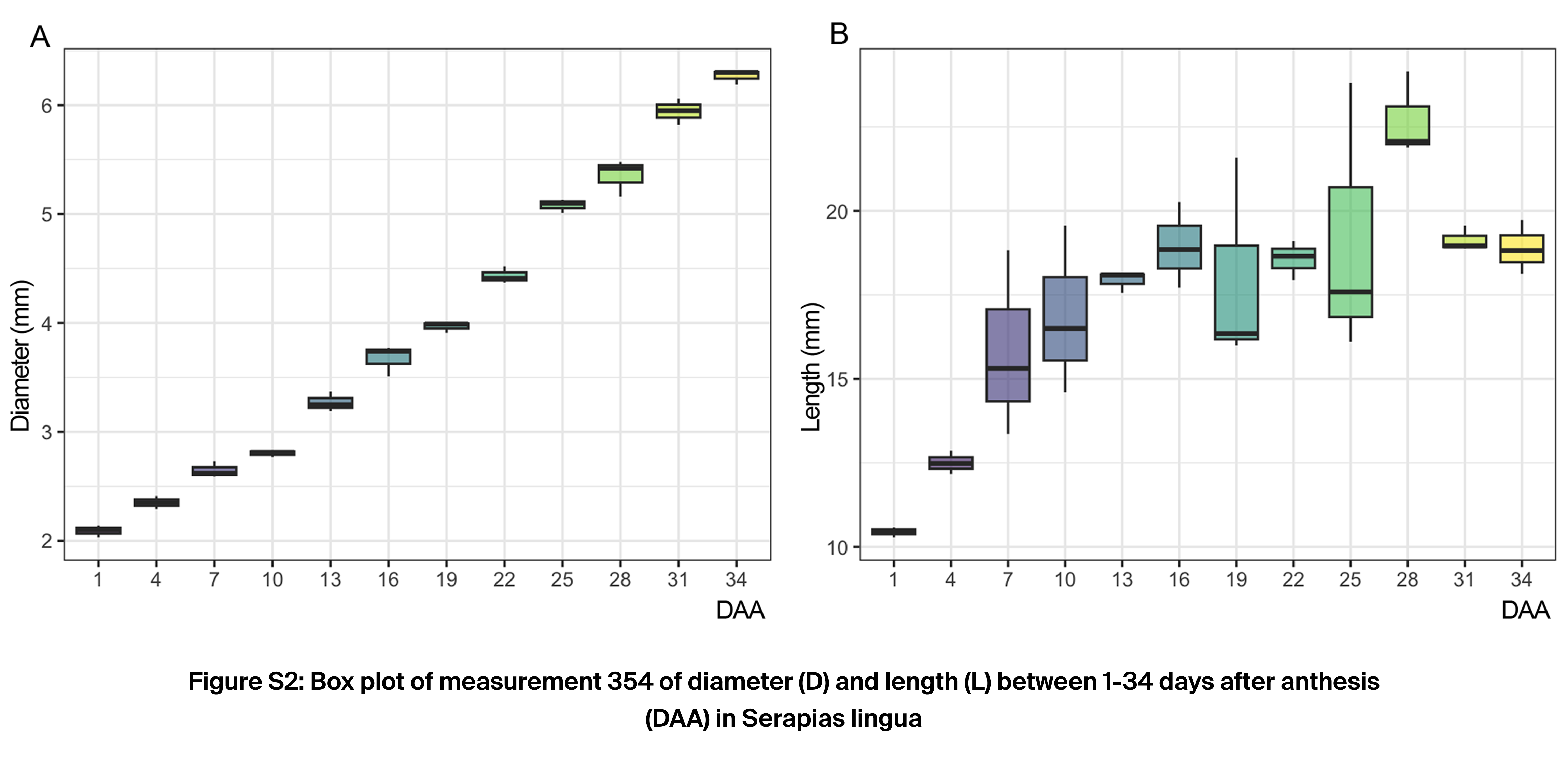

Supplement: Supplementary file 1 [file plants-14-01229-s001.zip › S2.png]

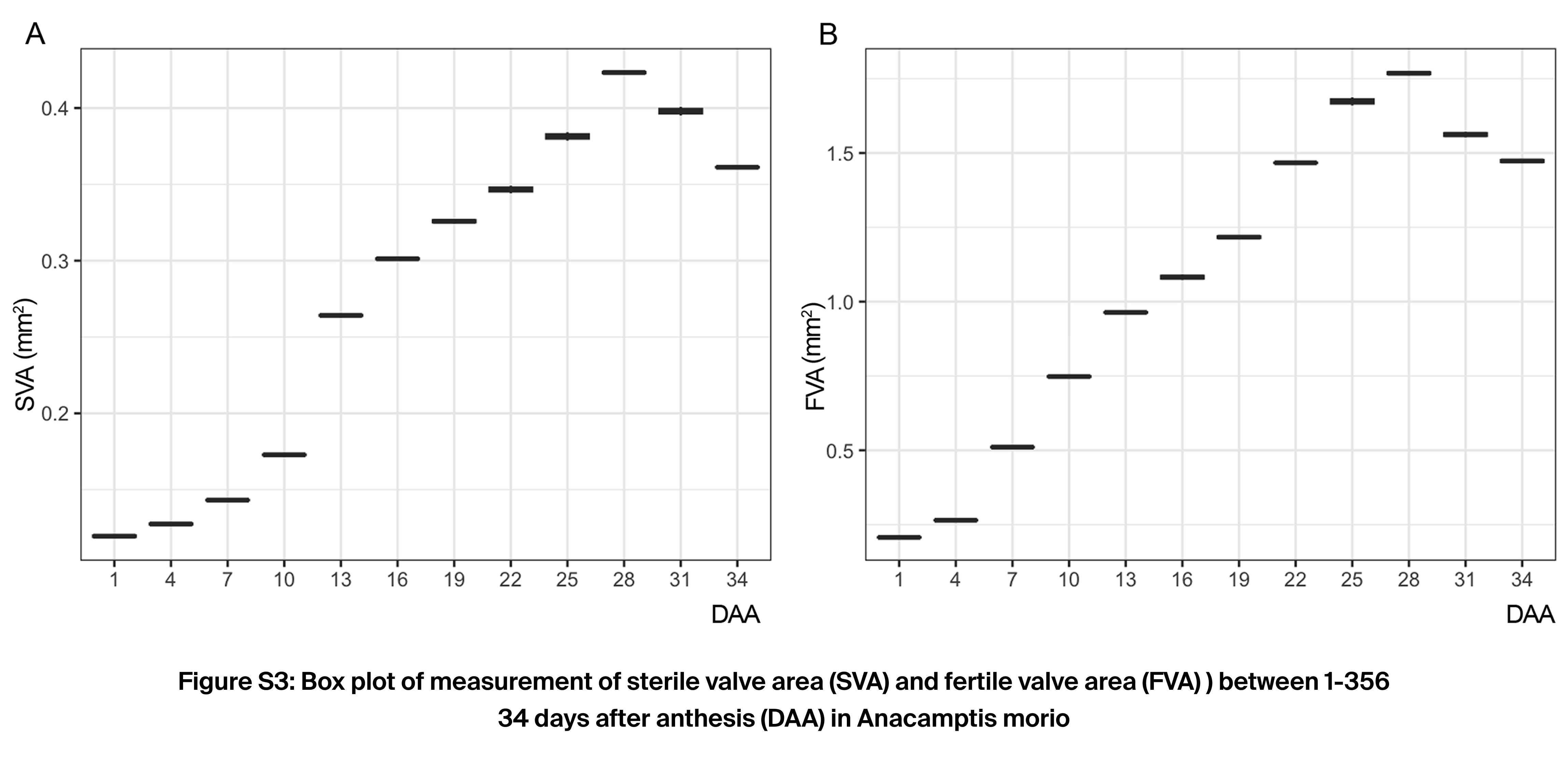

Supplement: Supplementary file 1 [file plants-14-01229-s001.zip › S3.png]

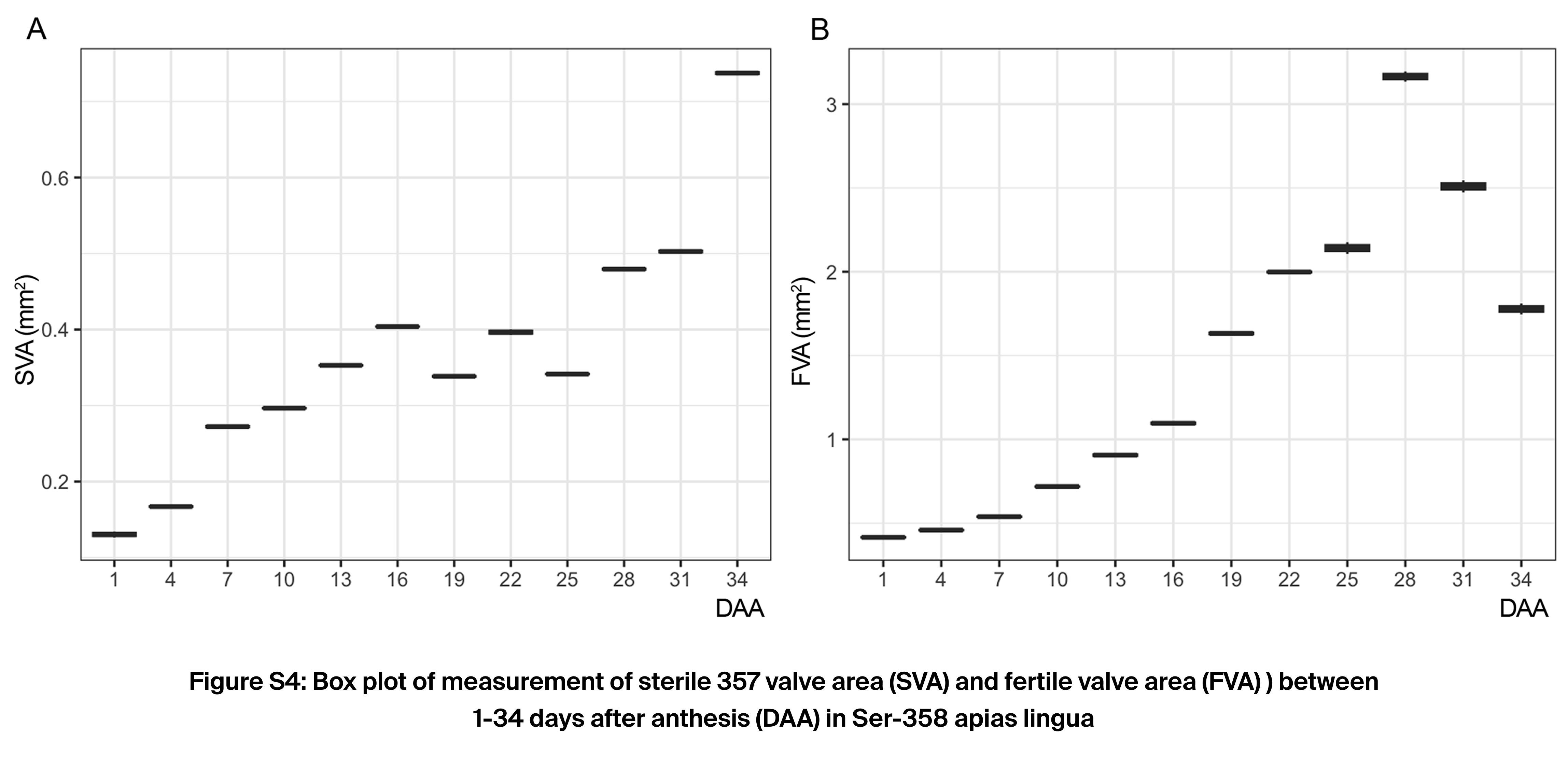

Supplement: Supplementary file 1 [file plants-14-01229-s001.zip › S4.png]
